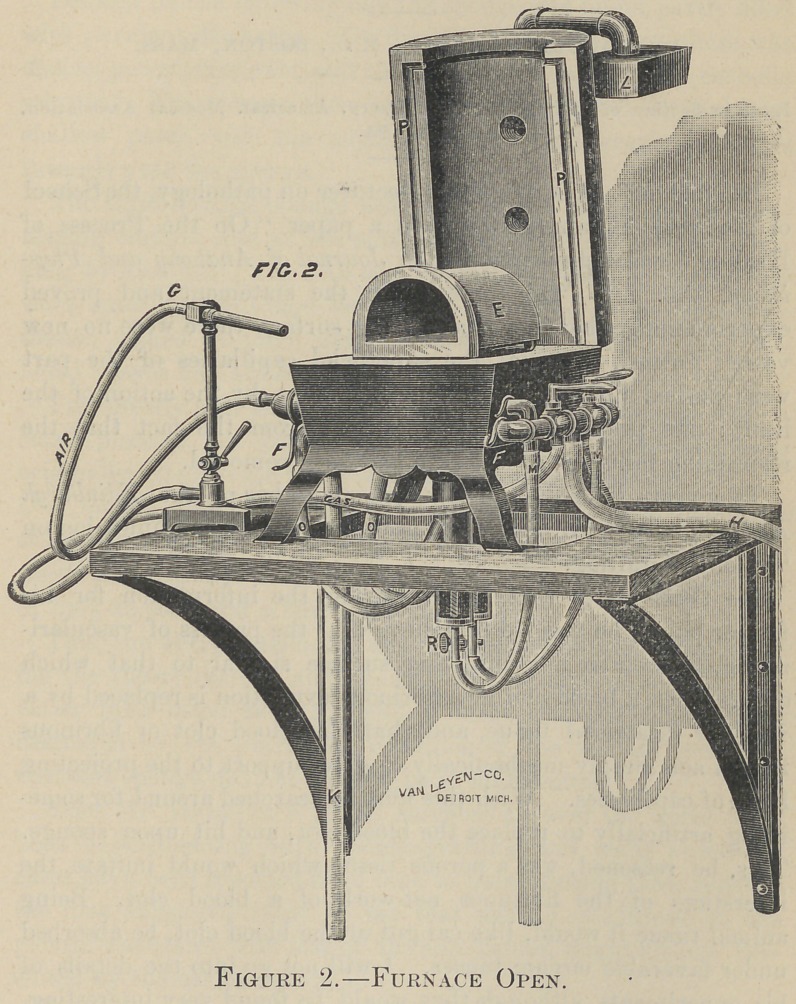# Description of Furnace

**Published:** 1885-03

**Authors:** C. H. Land

**Affiliations:** Dentist, Detroit


### Description of Furnace.

BY C. H. LAND, DENTIST, DETROIT.

Fig. 1* illustrates the furnace closed and ready for muffle work; AA is iron pipe, capable of both a sliding and swinging motion—see L Fig. 2; observe that it is connected to rubber tubing B, and with pipe having an air cock C, which regulates the quantity of air passing into the mouth of the muffle; it will also be noticed that this pipe passes over the two holes DD; thus by the escaping flame the pipe is heated to redness, and provides a superheated air before reaching the muffle. This column of air forced into the muffle keeps up a counter pressure within so much greater than the pressure produced by the blast within the fire chamber that all foul gases are prevented from entering the muffle, even though it be cracked.

Fig. 2 illustrates the furnace thrown open, being swung on hinges at the back, exposing the muffle E. The groove, PP, is packed with asbestos fibre, so that when the sections are brought together the furnace will be perfectly air and gas tight. The hooks, FF, are to hold the upper section secure to the lower. The gas and air connections are so arranged that the ordinary blow pipe can be attached, as shown at G. When the muffle E is removed it exposes two burners and a fire-brick surface made to fit the various appliances for crucible, forge, ladle, and blow pipe work. One or both burners can be operated in connection with the blowpipe. The air cock R is to provide a means of shutting off the air supply from either burner when required. H is the gas supply, K, air pipe connecting with the bellows No. 9 B. Buffalo Dental Mfg. Co.'s make. Size of muffle inside measurement, 8 inches long, 3½ wide, 2½ high. With gasoline vapor enameling can be done in from ten to fifteen minutes. Ordinary city gas in from fifteen to twenty-five minutes, according to quality. In thirty minutes a heat sufficient to destroy the muffle can be produced which indicates a temperature of over 3240° F, much higher than is ever needed for any kind of porcelain work, and will melt wrought iron.

(* See cuts on succeeding pages.)

## Figures and Tables

**Figure 1. f1:**
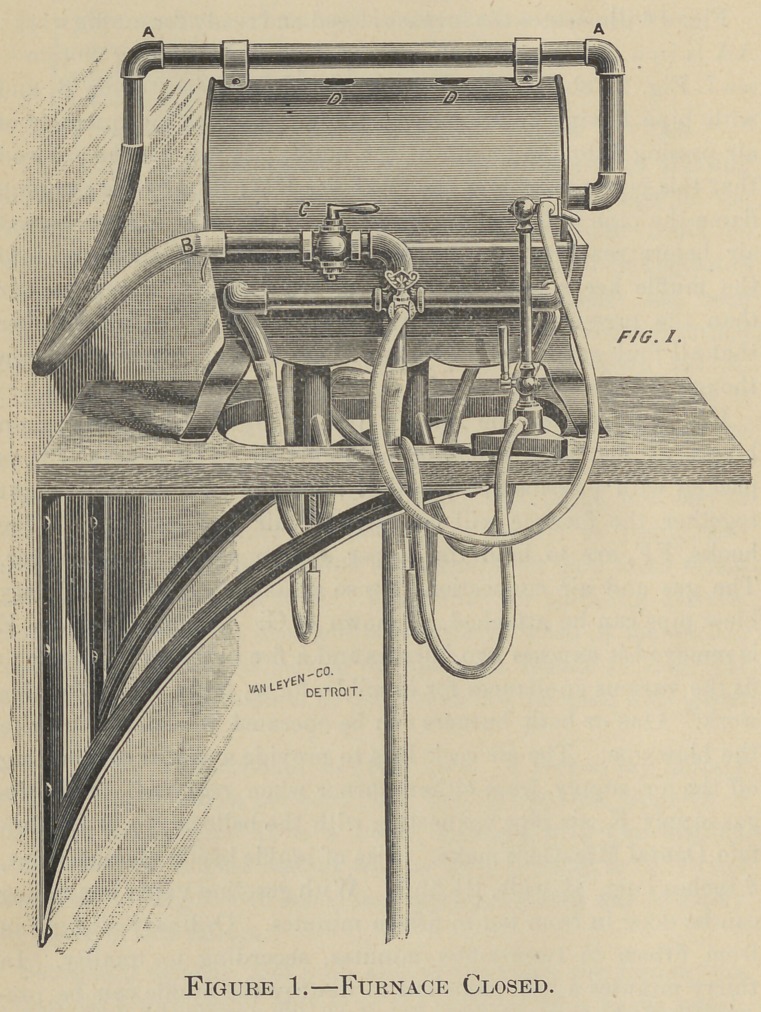


**Figure 2. f2:**